# TRPV1-Estradiol Stereospecific Relationship Underlies Cell Survival in Oxidative Cell Death

**DOI:** 10.3389/fphys.2020.00444

**Published:** 2020-05-26

**Authors:** Ricardo Ramírez-Barrantes, Karina Carvajal-Zamorano, Belen Rodriguez, Claudio Cordova, Carlo Lozano, Felipe Simon, Paula Díaz, Pablo Muñoz, Ivanny Marchant, Ramón Latorre, Karen Castillo, Pablo Olivero

**Affiliations:** ^1^Laboratorio de Estructura y Función Celular, Escuela de Medicina, Facultad de Medicina, Universidad de Valparaíso, Valparaíso, Chile; ^2^Escuela de Tecnología Médica, Universidad Andrés Bello, Viña del Mar, Chile; ^3^Centro Interdisciplinario de Neurociencia de Valparaíso, Facultad de Ciencias, Universidad de Valparaíso, Valparaíso, Chile; ^4^Centro Interoperativo en Ciencias Odontológicas y Médicas, Universidad de Valparaíso, Valparaíso, Chile; ^5^Facultad de Ciencias de la Vida, Universidad Andrés Bello, Santiago, Chile; ^6^Millennium Nucleus of Ion Channels-Associated Diseases (MiNICAD), Universidad de Chile, Santiago, Chile; ^7^Centro de Neurología Traslacional, Facultad de Medicina, Universidad de Valparaíso, Valparaíso, Chile

**Keywords:** TRPV1, 17β-estradiol, cell death, membrane receptor, neuroprotection

## Abstract

17β-estradiol is a neuronal survival factor against oxidative stress that triggers its protective effect even in the absence of classical estrogen receptors. The polymodal transient receptor potential vanilloid subtype 1 (TRPV1) channel has been proposed as a steroid receptor implied in tissue protection against oxidative damage. We show here that TRPV1 is sufficient condition for 17β-estradiol to enhance metabolic performance in injured cells. Specifically, in TRPV1 expressing cells, the application of 17β-estradiol within the first 3 h avoided H_2_O_2_-dependent mitochondrial depolarization and the activation of caspase 3/7 protecting against the irreversible damage triggered by H_2_O_2_. Furthermore, 17β-estradiol potentiates TRPV1 single channel activity associated with an increased open probability. This effect was not observed after the application of 17α-estradiol. We explored the TRPV1-Estrogen relationship also in primary culture of hippocampal-derived neurons and observed that 17β-estradiol cell protection against H_2_O_2_-induced damage was independent of estrogen receptors pathway activation, membrane started and stereospecific. These results support the role of TRPV1 as a 17β-estradiol-activated ionotropic membrane receptor coupling with mitochondrial function and cell survival.

## Graphical Abstract

TRPV1 is sufficient condition for 17β-estradiol resistance against oxidative stress-induced cell death. The cell death induced by oxidative stress could be characterized by a kinetic model of three state: alive, vulnerable and dead. In particular, the transition from alive to vulnerable state involves the depolarization of mitochondrial membrane potential, and the evolution to cell death the activation of caspases. Only in TRPV1 expressing cells, the initial application of 17β-estradiol protects against the irreversible damage triggered by oxidative stress, by inhibition of the transition from alive to vulnerable state. This mechanism includes blocking oxidative stress-dependent mitochondrial depolarization and the activation of caspases 3/7. Furthermore, 17β-estradiol potentiates TRPV1 activity associated with an increased open probability. Despite the relationship between the activation of TRPV1 and the maintenance of the mitochondrial membrane potential should be clarified, it could include the increase of the calcium buffer capacity of the mitochondria and/or the overexpression of anti-apoptotic proteins such as the Bcl-2 family as previously reported. Finally, the 17β-estradiol cell protection was independent of estrogen receptors, and was membrane started and stereospecific. These results support the role of TRPV1 as a 17β-estradiol ionotropic receptor being critical to cell survival.

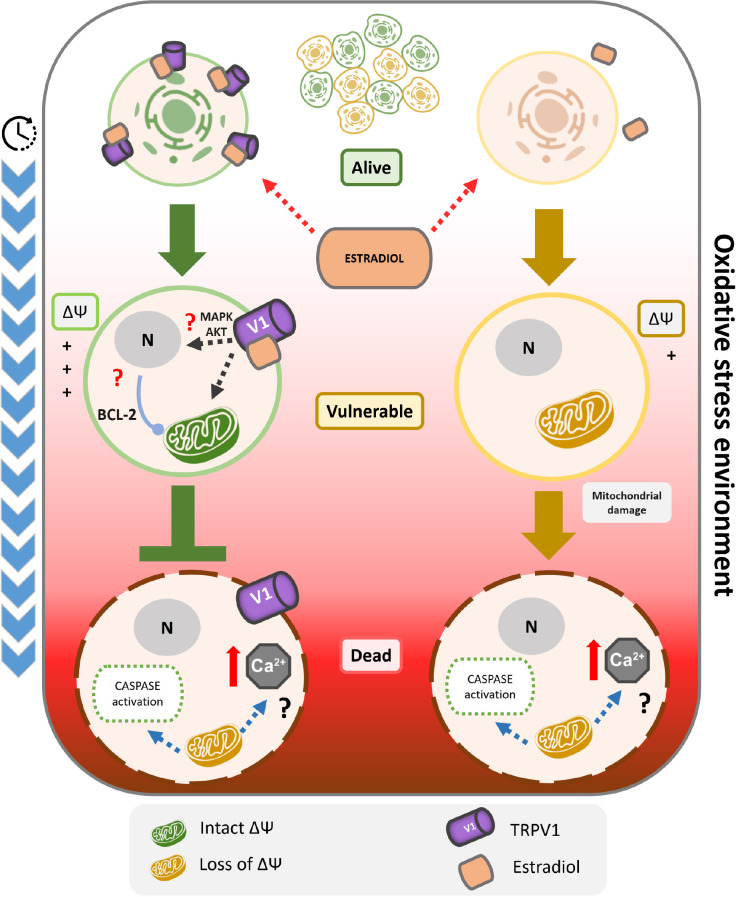

## Introduction

Oxidative dynamics is involved in several physiological processes, and disruption of redox control is a general pathological condition that induces cell dysfunction and death (Ghezzi et al., 2017). Oxidative stress is involved in several chronic conditions such as Parkinson’s disease and Alzheimer’s disease (Islam, 2017) and also in acute injuries as stroke and ischemia/reperfusion, damaging damaging several organs (Rodrigo et al., 2013). In this sense, aromatization of testosterone to estradiol is not restricted to classical endocrine tissues and has been associated with neurogenesis and tissue response to injury (Tenkorang et al., 2018; Duncan and Saldanha, 2019). Apart from classical steroid mechanism of action, a large body of evidence nowadays shows a new mechanism which appear to involve membrane-associated signaling complexes (Wu et al., 2005, 2011). Such responses could be independent or in conjunction with estrogen receptors α and β, suggesting that estrogens such as 17β-estradiol modulate neural function by direct effects on membrane receptors (Balthazart and Ball, 2006; Vega-Vela et al., 2017). These “membrane actions” of estrogen involve mainly ionic channels conduction and permeation regulation, kinase activation and transient increase of intracellular Ca^2+^. These effects can trigger different signaling pathways that are critical for regulation of plasticity, cognition, neuroprotection and maintenance of homeostasis. Moreover, this extra-nuclear action has been shown to be critical for protection against oxidative stress-induced cell death. For instance, it has been demonstrated that estrogen-induced rapid Ca^2+^ influx, via the voltage-gated L-type Ca^2+^ channel is key to initiate the downstream Src/ERK signaling pathways leading to estrogen neuroprotection through activation of the transcription factor CREB and subsequent increase of Bcl-2 expression in hippocampal neurons (Wu et al., 2005). Also, this Ca^2+^ himself triggered by estrogen could induce an increase in mitochondrial Ca^2+^ sequestration and promote mitochondrial tolerance against glutamate excitotoxicity in hippocampal neurons.

In this context, the polymodal TRP ion channels have emerged as potential targets of membrane signaling of steroids, in particular, those of the melastatin (M) and vanilloid (V) families (Kumar et al., 2015). TRPM3 was the first described steroid-sensitive ionotropic receptor shown to be rapidly and reversibly activated by pregnenolone sulfate, inducing transient calcium influx (Wagner et al., 2008; Thiel et al., 2013). Additionally, 17β-estradiol triggers a physiological rapid intracellular calcium response, via Ca^2+^ influx through TRPV5 and TRPV6, during transepithelial Ca^2+^ transport (Irnaten et al., 2008, 2009; Kumar et al., 2017). In particular, 17β-estradiol modulates TRPV1 expression and activity in cervical afferent neurons, in dorsal root ganglion cells and hippocampus (Tong et al., 2006; Wu et al., 2010; Cho and Chaban, 2012; Pohóczky et al., 2016; Yamagata et al., 2016) controlling pain sensation and tissue viability, through fine regulation of calcium homeostasis. It has been recently demonstrated that the TRPV family located at the central nervous system (hypothalamus, hippocampus, cortex, brainstem) has estrogen receptor binding sites that are inducible by gene promoters, whose expression may be regulated by the estrous cycle (Kumar et al., 2018).

The modulation of TRP family by estrogens could be relevant due to the variety of roles that TRPs play in excitable and non-excitable cells, accounting for sensory physiology, proliferation, growth, male fertility, neuronal plasticity and regulation of oxidative stress-induced cell death. The function of TRPV family and particularly TRPV1 member in cell death merits special consideration. With a still unclear role in cell viability, TRPV1 non-selective cation channel is able to integrate physicochemical stimuli such as temperature, voltage, proton gradients and osmolarity (Caterina et al., 1997; Nishihara et al., 2011; Canul-Sánchez et al., 2018). Micromolar concentrations of the classical TRPV1-agonist capsaicin (CAP) and acid solution (pH 5.5) induce cytosolic calcium increase, ROS production, mitochondrial membrane depolarization and cell death (Hu et al., 2008). In rat cortical neurons TRPV1 activation by CAP induces apoptotic cell death via L-Type Ca^2+^ channels, generating Ca^2+^ influx, ERK phosphorylation, ROS production and caspase-3 activation (Shirakawa et al., 2008). However, similar results have been reported for CAP without TRPV1 participation, suggesting both dependent and independent effects of this vanilloid (MacHo et al., 2000). Our explanation is that the amount of CAP is able to modify the amount of calcium entry and release from inner cell stores (Ramírez-Barrantes et al., 2018).

The regulation of the channel might be critical for maintaining cellular homeostasis in oxidative environment. TRPV1 *knock-out* (*KO*) mice have estrogen sensitive tissues like testis, much more sensitive to cell death by oxidative stress stimuli (Mizrak and van Dissel-Emiliani, 2008). Moreover, in hippocampus subjected to 10 min ischemia, CA1 neurons pre-treated with CAP are less sensitive to cell death and the effect is inhibited by the TRPV1 antagonist capsazepine (CPZ). The mechanism suggested involves a moderate increase in Ca^2+^ via TRPV1. This transient Ca^2+^ influx may induce tolerance to the subsequent calcium overload, preconditioning the response and inducing neuroprotection (Pegorini et al., 2005; Huang et al., 2017). Also, in rats, the activation of TRPV1 by CAP in substancia nigra pars compacta is able to diminish cell death triggered by MPP, via reduced activation of microglia and decrease of ROS levels (Park et al., 2012).

This paradoxical effect of TRPV1 points the importance of the channel in cell survival, choosing the activation of different responses depending on the cell context, the moment of activation, and transience of the signal.

The ability of estrogen to modulate the expression and function of TRPV1 channel may imply a specific mechanism to control cellular homeostasis. Cholesterol, pregnenolone and testosterone can inhibit TRPV1-mediated currents by different ways. However, 17β-estradiol is the only steroid able to modulate the activation of the channel during enhanced CAP-evoked current in dorsal root ganglion neurons (Chen et al., 2004) and in CAP-induced nociception (Lu et al., 2009). Although the differential role of 17β-estradiol, an aromatic steroid, in allosteric modulation of TRPV1 is unclear, aromatization seems to convert an inhibitory steroid to an excitatory one. Then, can steroids differentially modulate cell viability through TRPV1? and can be the aromatic capacity relevant for paracrine and autocrine cellular protection against oxidative cell death? Here, we show that 17β-estradiol and not testosterone or 17α-estradiol, induced cell protection via modulation of TRPV1 activity during oxidative injury independently of estrogen receptor expression.

## Methods

### Cell Culture

HeLa cells were obtained from ATCC (Manassas, VA, United States). We used culture medium DMEM (Dulbecco’s Modified Eagle medium) supplemented with 10% fetal bovine serum (FBS) and 50 U/mL of penicillin-streptomycin. We incubated cells in conventional incubator at 37°C, in steam saturated 95% air, 5% CO_2_ atmosphere.

### TRPV1 Stable Line Construction

Transfections were performed using DNA: Transit IT-LT1 (Mirus Bio LLC, Madison, United States) at a ratio of 1:3 according to the manufacturer’s protocol. Cell dishes were transfected with the pCDNA3.1-TRPV1. We selected the HeLa cells 48 h after transfection using Geneticin (Sigma-Aldrich, St. Louis, MO, United States, 800 mg/mL). Cells were maintained at this Geneticin concentration in all experiments. Time course of stable TRPV1 expression was followed by PCR once a week.

### Reverse Transcription PCR (RT-PCR)

Total RNA from both parental and transfected TRPV1 cell lines was extracted with Trizol (Invitrogen, Carlsbad, CA, United States). cDNA libraries were generated by RT-PCR using M-MLV reverse transcriptase (Invitrogen, Carlsbad, CA, United States). Equal amounts of RNA were used as templates in each reaction. The RT-PCR product for TRPV1 generated a 167 bp amplicon. The other targets had the following sizes: estrogen receptor α, 153 bp; estrogen receptor β, 139 bp; aromatase, 134 bp; and S16, 102 bp. All of the samples were simultaneously amplified with appropriate primers and annealing temperature. The PCR reaction was performed using the Go Taq master mix (Promega Corp., Madison, WI, United States) containing all of the reagents for the amplification reaction except for the cDNA template. The protocol consisted in denaturation at 94°C for 5 min, followed by 40 cycles of: 30 s denaturation at 94°C, 30 s annealing at 55–58°C and 30 s extension at 72°C. The final elongation was performed at 72°C for 10 min, and the samples were held at 4°C once the final PCR step was completed. The PCR products and ladder (New England Biolabs, United Kingdom) were loaded onto a 2% agarose gel (Lonza Rockland, ME, United States), electrophoresed and stained with ethidium bromide (Merck KGaA, Darmstadt, Germany).

### Calcium Signal Recordings

Cell cultures were loaded with Fura-2AM (Molecular Probes, Eugene, OR, United States) for 30 min at room temperature in extracellular solution containing 130 mM NaCl, 5.4 mM KCl, 2.5 mM CaCl_2_, 0.8 mM MgCl_2_, 5.6 mM glucose, and 10 mM HEPES, pH 7.4 (adjusted with Tris base). The cells were then rinsed and allowed to equilibrate for 5–10 min. CAP-induced Ca^2+^ activity was recorded by epifluorescence microscopy using an Olympus IX81 microscope (Olympus, Japan) equipped with dual-excitation wavelength with a minimum recording time of 2 s for Fura 2. The maximum resolution was obtained using objective lens Olympus Plan Apo X40 oil 1.3 NA. We calculated the concentration of cytosolic calcium from the recorded fluorescence intensity using the following equation:

$$\left[C\,a^{2+}\right]=K\,d\times \left[\frac{R-R_{\min}}{R_{\max}-R}\right]\times \frac{S\,f}{S\,b}$$

where *Kd* is the Fura 2 dissociation constant at 37°C (224 nM), *R* is the ratio of fluorescence measured at 340 and 380 nm, respectively, and *Sf/Sb* is the 380 nm ratio of fluorescence in low-calcium buffer referred to high-calcium buffer.

### Animal Experimentation

This study was carried out in accordance with the principles of the Basel Declaration and recommendations of the National Institute of Health (USA) and performed in strict accordance with the recommendations of the Guide for the Care and Use of Laboratory Animals of the Ethics Committee for Animal Experimentation Committee as well as the Biosecurity Committee of the University of Valparaíso. All of the animals were handled according to approved institutional animal care and used committee protocols (BEA125-18) of the University of Valparaiso. All surgery was performed under tricaine anesthesia, and every effort was made to minimize suffering.

### Heterologous Expression System

*Xenopus laevis* oocytes were used to measure TRPV1 currents. mMESSAGE mMACHINE from Ambion (Waltham, MA, United States) was used for *in vitro* transcription of the cRNA of wild type TRPV1 rats (GenBank^TM^ accession no. NM031982). The oocytes were injected with 3 ng of cRNA and then incubated in ND96 solution (in mM: 96 NaCl, 2 KCl, 1.8 CaCl2, 1 MgCl_2_, 5 HEPES, pH 7.4) at 18°C for 3–5 days before electrophysiological recordings.

### Electrophysiological Recordings

Macroscopic and single channel current recordings were made employing the patch-clamp technique with the cell-attached and inside-out configurations, respectively. Symmetrical recording solutions contained: 150 mM NaCl, 10 mM EGTA, 2 mM MgCl_2_, 10 mM HEPES, pH 7.4. 17β-estradiol (E2) and other hormones were prepared in recording solutions at the final concentrations indicated, and perfused into the recording chamber, exchanging at least 10-times the chamber volume. Data were acquired with an Axopatch 200B amplifier (Molecular Devices), and the Clampex 10.7 acquisition software (Molecular Devices). Both the voltage command and current output were recorded at 100 kHz and filtered at 20 kHz using an 8-pole Bessel low-pass filter (Frequency Devices) and sampled with a 16-bit A/D converter (Digidata 1550B; Molecular Devices). Borosilicate capillary glasses (1B150F-4, World Precision Instruments, Sarasota, FL, United States) were pulled in a horizontal pipette puller (Sutter Instrument, Novato, CA, United States) and fire-polished with a microforge (MF-830, Narishige, Tokyo, Japan). All experiments were performed at room temperature (20–22°C). Macroscopic current recordings were evoked by pulses of –100 to +350 mV in 20 mV increments, with pulses of decreasing duration as potential increases, followed by a step at 190 mV, to obtain the tail currents.

### Cell Death Protocols

Cells were exposed to the experimental conditions in DMEM supplemented with 1% bovine serum albumin instead of FBS. H_2_O_2_ (Merck KGaA, Darmstadt, Germany) was added to cell cultures for 24 h in the absence or presence of the following drugs: CAP, CPZ, 17β-estradiol, 17α-estradiol (Tocris Bioscience, Bristol, United Kingdom), 17β-estradiol-BSA and testosterone (Sigma-Aldrich, St. Louis, MO, United States). After 24 h, the cultures were stained with Rhodamine 123 (Rhod 123; Invitrogen, Carlsbad, CA, United States; 100 nM) and propidium iodide (PI; Sigma-Aldrich, St. Louis, MO, United States; 10 μg/mL), JC-1 (Invitrogen, Carlsbad, CA, United States, 2 μM), or cell event caspase 3/7 (Invitrogen, Carlsbad, CA, United States).

### Three State Model Evaluation by Flow Cytometry

We interpreted our results using a three-state, alive (A)-vulnerable (V)-dead (D) model. To quantify the three cellular states, mitochondrial function and plasma membrane integrity were recorded over time using Rhod 123 and PI fluorescence intensity. Mitochondrial membrane potential (Δψ) was monitored using Rhod 123 in non-quenching mode. Rhod 123 is a fluorescent membrane-permeant cation, which passively distributes across membranes according to the membrane potential. In non-quenching mode, mitochondrial depolarization causes Rhod 123 efflux from the mitochondrial matrix into the cytosol resulting in a decrease of fluorescence intensity. Depolarized mitochondria will have lower cationic dye concentrations and lower fluorescence, while hyperpolarized mitochondria will have higher dye concentrations and fluorescence. To calibrate Rhod 123, we performed a temporal course of mitochondrial depolarization using the mitochondrial uncoupler carbonyl cyanide 4-(trifluoromethoxy) phenylhydrazone (FCCP, 10 μM) by flow cytometry. We determined that a 15 min exposition to Rhod 123 at 0.5 μg/mL was sufficient to measure the mitochondrial depolarization in non/quenching mode and applied this strategy in each experiment. To measure the integrity of plasma membrane, we used propidium iodide (PI, 10 μg/mL), a DNA intercalating that binds to cellular DNA when plasma membrane integrity is lost. We recorded the time course of cell death in ethanol 10% to determine the maximum signal of PI. Each experiment was accompanied with an ethanol control.

The A-V-D model distinguishes three states from the fluorescence intensity of Rhod 123 and PI. The alive state corresponds to cells with high fluorescence intensity for Rhod 123 and low PI fluorescence intensity, which indicate, respectively, optimal function of mitochondrial membrane potential and impermeability of plasma membrane. Conversely, the dead state identifies cells with low Rhod 123 fluorescence intensity and high PI fluorescence intensity, indicating a fall in the mitochondrial membrane potential and the permeabilization of the plasma membrane. Finally, the vulnerable state corresponds to cells with one of these two parameters altered. Cells were measured by flow cytometry (FACScalibur, BD, Biosciences, CA, United States). We acquired a minimum of 10,000 cells in each experiment and excluded from the analysis debris and duplets. The analysis was performed using FlowJo software (Tree Star Inc., Ashland, OR, United States). To calculate the cell fraction or probability of each state, the data were normalized using the following formula:

$$X_{\left(A-V-D\right)}=\frac{n^{{}^{\circ}}\,x}{n^{{}^{\circ}}\,\left(A+V+D\right)}$$

Where, X is cell state fraction or probability, n°x the number of cells in state x, and n°(A + V + D) total number of cells.

### Measurement of Mitochondrial Membrane Potential (ΔΨm)

JC-1, a sensitive fluorescent probe for ΔΨm, was used (Invitrogen, Carlsbad, CA, United States) after specific experimental procedure. Parental HeLa cells and st-TRPV1 were rinsed twice with PBS and stained with 2 μM JC-1 for 30 min at 37°C. Cells were rinsed twice with PBS and immediately analyzed by FACScalibur flow cytometer (BD, Biosciences, CA, United States). We used a 488 nm excitation filter, a 530 nm emission filter (FL1) and a 585 nm emission filter (the fluorescence 2: FL2). A logarithmic transformation was applied to the values of photomultiplier. Green fluorescence (FL1) represents the monomeric form of JC-1 corresponding to the mitochondrial mass. Red-orange fluorescence (FL2) corresponds to the J-aggregate form of JC-1. The analysis was performed using FlowJo software (Tree Star Inc., Ashland, OR, United States). Mitochondrial depolarization was indicated by an increase in the red/green fluorescence intensity ratio.

### Caspases-3/7 Activity Measurement

Cell Event^TM^ assay (Invitrogen, Carlsbad, CA, United States) was used to measure the activity of caspase-3/7 enzymes. After specific experimental procedure Parental HeLa cells and st-TRPV1 cells were collected, and the reagents were incubated for 30 min. The fluorescent intensity (at 485 nm excitation and 535 nm emission) was monitored with FACScalibur cytometer, BD, Biosciences, CA, United States) using 488 nm laser of excitation and FL1 emission filter (530/30 bp). The analysis was performed using FlowJo software (Tree Star Inc., Ashland, OR, United States).

### Primary Hippocampal Cultures

Pregnant *Sprague Dawley* rats were handled under standard conditions of temperature (12 h light/dark cycle) and *ad libitum* feeding, according to the guidelines of the Animal Care Committee of the University of Valparaíso (CICUAL-UV). Hippocampi were isolated at embryonic day 18 and washed with Hanks saline solution containing in mM (135 NaCl, 5.4 KCl, 0.5 NaH_2_PO_4_, 0.33 Na_2_HPO_4_, and 5.5 D-glucose) balanced at pH 7.4 at 4°C. They were trypsinized and mechanically disaggregated into MEM 10 (MEM, 19.4 mM D-glucose, 26 mM NaHCO_3_, supplemented with 10% horse serum, 10 U/mL penicillin, 10 μg/mL streptomycin). The non-disintegrated tissue was centrifuged at 800 rpm for 10 s. The cell suspension was seeded in MEM 10 at a density of 40,000 cells per 12 mm diameter glass cover previously treated with poly-lysine (50 μg/mL) and kept in a humid atmosphere, saturated with 5% CO_2_. After 1 h, MEM 10 was replaced by serum-free neurobasal medium supplemented with B27 and 2 mM GlutamaxTM (Invitrogen, Carlsbad, CA, United States). Cells were used at 11–14 DIV (Muñoz et al., 2011).

### Immunofluorescence

Adult rats (1 month) were transcardially perfused with 4% paraformaldehyde (PFA) in phosphate buffer. The brain was equilibrated in 30% sucrose solution, embedded in cryopreservant (OCT) and sectioned at 20 μm using a cryostat (Leica CM1900). Floating cuts were incubated in permeabilization/blocking buffer (0.7% Triton X-100, 0.1% sodium borohydride and 10% goat serum in PBS) overnight at 4°C. Sections were washed and incubated with primary rabbit polyclonal antibody against TRPV1 (dilution 1: 200, abcam, Cambridge, MA, United States) and with primary mouse monoclonal antibody against anti-β-Tubulin III (1: 500, Millipore) overnight at 4°C in PBS-TX (0.7% Triton X-100 and 10% goat serum in PBS). The slices were then washed and incubated for 2 h with donkey-antirabbit Alexa Fluor 546 and donkey-antimouse AlexaFluor 488 antibodies (1: 500), obtained from Molecular Probes. Hoechst^®^ 33342 was used as nuclei marker according to the manufacturer’s instructions (Molecular Probes). Images were obtained using a confocal microscope (Nikon Eclipse C180i).

For immunofluorescence in cultured neurons, we used a similar protocol to that previously described, with the exception that the cells were fixed directly by incubation with 4% PFA and 4% sucrose for 40 min and subsequently blocked in solution without borohydride.

### Neuronal Viability Determined Using 4, 5-Dimethylthiazol-2-yl)-2, 5-Diphenyl Tetrazolium (MTT)

MTT assay was used to evaluate the reduction-oxidation status of living cells and mitochondrial activity, reflecting cell survival due to the formation of formazan. A density of 1 × 10^4^ cells/well in 96-well plates was used for the MTT assay. Briefly, after treatment neurons were incubated in medium containing 500 μg/mL MTT for 3 h at 37°C. MTT medium was removed by plate inversion and 100 μL DMSO was added to each well to dissolve the formazan crystals. The plates were read using an Anthos2020 microplate reader at a wavelength of 570 nm and a reference of 690 nm.

### Data Analysis

All the results are presented as mean ± S.D. from at least three independent assays for each experimental condition. Data were analyzed with Origin Pro (OriginLab Corporation Northampton, United States). We compared multiple groups with the Fisher’s least significant difference procedure and ANOVA followed by the Bonferroni *post hoc* test in Statgraphics Plus 5.0 (GraphPad Software, Inc., San Diego, CA, United States). The results were considered statistically significant with *P* < 0.05. All electrophysiology data analyses were performed with Clampfit 10.7 (Molecular Devices), GraphPad Prism 6, and Excel 2013 (Microsoft, Redmont, WA, United States). Tail currents were used to build the G-V relationships, fitted with a Boltzmann function: G = Gmax/(1-exp^(–*zF(V*–*V*0.5)/RT)^), where Gmax is the maximum conductance, z is the voltage dependence of activation, V_0.5_ is the half-activation voltage, T is the absolute temperature, F is the Faraday’s constant and R is the universal gas constant. Gmax, V_0.5_, and z were determined by using the solver complement of Microsoft Excel. Data were aligned by shifting them along the voltage axis by the mean ΔV_0.5_ = (−V_0.5_), then binning them in a range of 25 mV, between –100 mV and up to 350 mV. Statistical analysis used a two-tailed Student’s *t*-test with a non-parametric *t*-test.

## Results

### 17β-Estradiol Enhanced TRPV1–Dependent Calcium Influx

**FIGURE 1:**
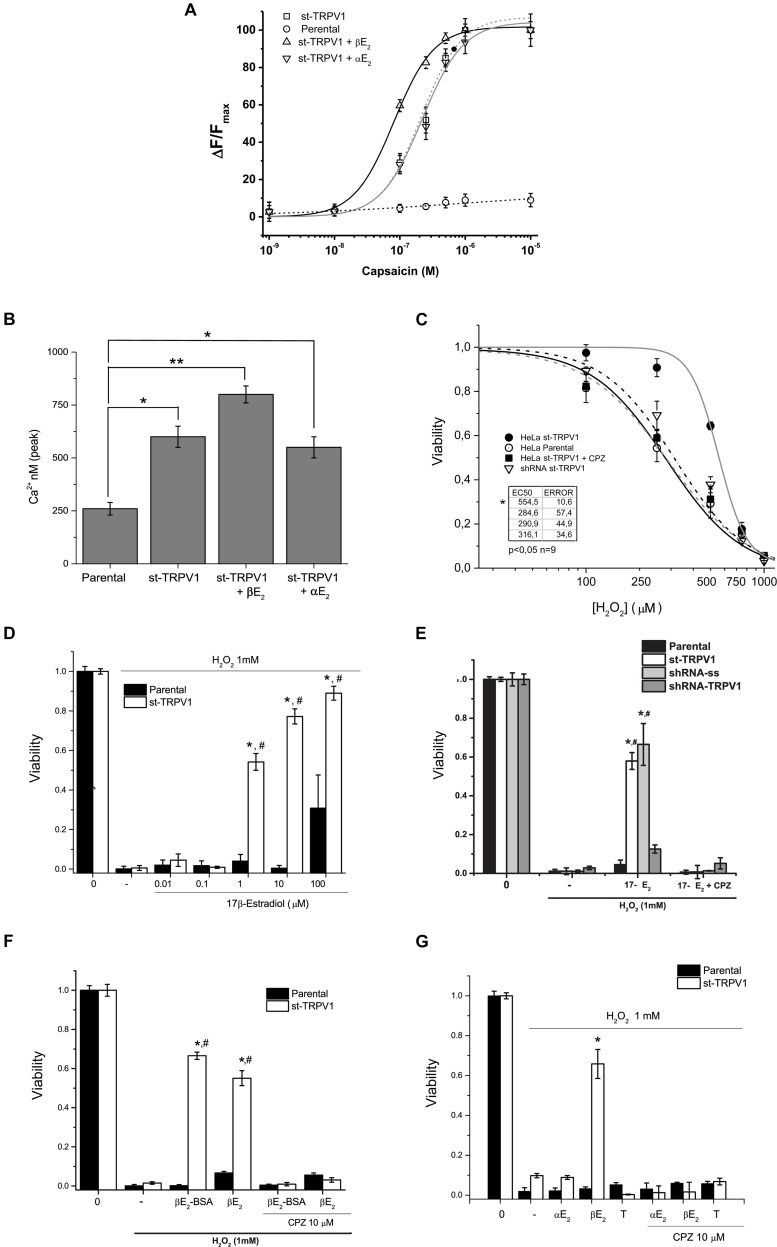
TRPV1 expression is necessary and sufficient condition to deploy specific 17β-estradiol protection against oxidative stress. **(A)** Fura2-AM calcium imaging of HeLa parental and HeLa st-TRPV1 in the presence or absence of 17β-/17α-estradiol exposed to different concentrations of capsaicin (M). **(B)** Calculated concentration of cytosolic calcium (nM) from the peak signal of previous recorded fluorescence. **(C)** Cell viability as a function of H_2_O_2_ (10^–5^–10^–3^ M) in parental and st-TRPV1 cells. (*N* = 9). **(D)** Dose-response bars graph of 17β-Estradiol (10^–8^–10^–4^ M) in parental (filled bars) and st-TRPV1 (empty bars) cells in presence of H_2_O_2_ (*N* = 9). **(E)** Bar graph of the effect of knocking-down TRPV1 (sh-RNA-TRPV1) on H_2_O_2_-induced cell death in presence of 17β-estradiol (*N* = 9, scramble shRNA, shRNA-ss). **(F)** H_2_O_2_-induced cell death (1 mM) in cells treated with 17β-estradiol (10^–6^ M) or 17β-estradiol-BSA (10^–6^ M). **(G)** Effect of H_2_O_2_ co-administered with steroids 17β-estradiol, 17α-estradiol (10^–6^ M) and testosterone (10^–6^ M). Graph bars show means ± SD. All the viability experiments were registered at 24 h and the results expressed as data normalized to untreated condition. Statistical differences were assessed by one-way analysis of variance followed by Bonferroni’s *post hoc* test. ^#^*P* < 0.05 vs. NT st-TRPV1, *P < 0.01 vs. parental cells.

In order to study the effect of 17β-estradiol in TRPV1 activity, parental cell cultures were stably transfected with pCDNA3.1-TRPV1 (st-TRPV1) and examined for TRPV1 functional expression. TRPV1 mRNA expression in st-TRPV1 was confirmed by RT-PCR that yielded a 104 bp amplicon in st-TRPV1 but not in parental cells. Immunofluorescence microscopy and flow cytometry also confirmed the expression of the protein, which was present in more than 80% of the cells (Supplementary Figure S1). To demonstrate that st-TRPV1 cells express a functional TRPV1, we performed functional analysis by means of the calcium imaging. st-TRPV1 showed a transient calcium influx after the exposition to 250 nM of the TRPV1 agonist CAP, followed by a decay to basal levels, even under sustained stimulation. Only st-TRPV1 cells were able to respond to several concentrations of CAP. TRPV1 activation saturated at 1 μM CAP. The dose-response CAP data were fitted using a Hill function finding an EC_50_ of 78 nM for st-TRPV1. Next, we studied the effect of 17β-estradiol in the TRPV1-dependent changes in intracellular Ca^2+^ concentration. The increase in calcium influx triggered by CAP was enhanced by 17β-estradiol (Fig. 1A) shifting the EC_50_ to 18 nM. The 17β-estradiol effect was completely inhibited by 10 μM CPZ. Additionally, st-TRPV1 incubated with 17α-estradiol failed to induce the increase of Ca^2+^ entry mediated by CAP (Fig. 1B), suggesting that estradiol enhances TRPV1-dependent intracellular Ca^2+^ concentration in a stereospecific manner.

### Specificity of 17β-Estradiol-Induced Cell Death Protection

To evaluate the role of TRPV1 in estrogen protection against oxidative stress-induced cell death, we utilized as inductor hydrogen peroxide (H_2_O_2_) and recorded simultaneously the mitochondrial function and plasma membrane integrity by flow cytometry applying the AVD model analysis (Ramírez-Barrantes et al., 2018). We performed a dose-response curve of H_2_O_2_ in both cell types and measured cell viability (Figure 1C). The mere expression of TRPV1 induced resistance to H_2_O_2_ injury at three different concentrations, but, at 1 mM H_2_O_2_ the cell dies independent of TRPV1 expression. The concomitant application of CAP (250 nM), however, favored the protective effect at 1 mM H_2_O_2__;_ this effect being abolished by CPZ (Supplementary Figures S2A,B).

In turn, 17β-estradiol exposure induced resistance to H_2_O_2_-induced cell death only in st-TRPV1 cells (Figure 1D). 17β-estradiol oxidative cell death protection was concentration-dependent and occurred through TRPV1 modulation (Figure 1D). In parental cells, 17β-estradiol had no protective effect, except for saturated concentration (100 μM) possibly via triggering a non-specific effect (Figure 1D). The protection elicited by 17β-estradiol was abolished using 10 μM CPZ reaching levels similar to controls (Figure 1E). None of the compounds used except for H_2_O_2_ modified the viability of HeLa cells (Supplementary Figure S3). Moreover, 17β-estradiol-mediated H_2_O_2_-induced cell death protection was significantly decreased by knocking down the expression of TRPV1 in st-TRPV1 cells with a shRNA-TRPV1 (Figure 1E). In addition, st-TRPV1 cells transfected with a scramble shRNA-SS did not show any difference compared with non-transfected cells (Figure 1E). This evidence strongly suggests that cell protection against oxidative stress by 17β-estradiol is mediated by TRPV1.

The Membrane-impermeable 17β-estradiol conjugated with albumin (17β-estradiol-BSA) was employed in order to test whether estrogen could exert its effects through membrane receptor. Interestingly, this probe preserved the cellular protection against H_2_O_2_ in st-TRPV1 cells, which was abolished by the TRPV1 antagonist CPZ (Figure 1F), supporting that a membrane receptor mediates the estrogen effect. To study whether the protective effect of 17β-estradiol was stereospecific, we carried out an experiment using 17α-estradiol, the 17β-estradiol stereoisomer. Parental and st-TRPV1 cells were exposed to 1 mM H_2_O_2_ in the absence or presence of 17β-estradiol, 17α-estradiol or testosterone and cell viability was tested by flow cytometry. 17α-estradiol (1 μM) was unable to protect against H_2_O_2_ (Figure 1G). Similarly, cells treated with testosterone did not show any protection against H_2_O_2_-induced cell death (Figure 1G). These results indicate that the TRPV1-dependent protection against H_2_O_2_-induced cell death is specifically mediated by 17β-estradiol.

### 17β-Estradiol Increased TRPV1 Activity

**FIGURE 2:**
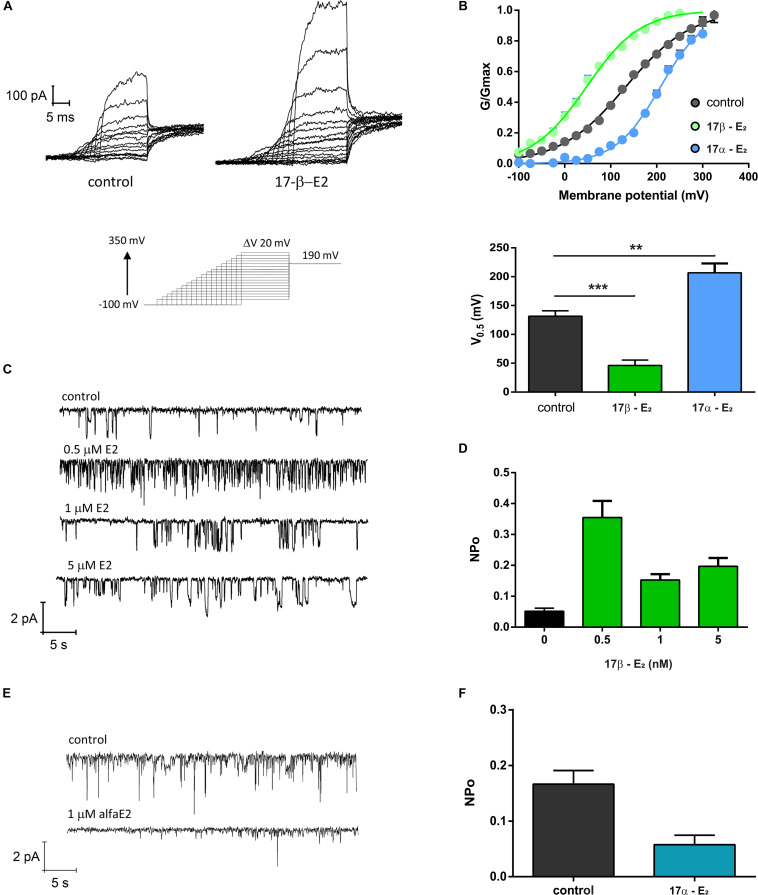
TRPV1 activity is enhanced by 17β-estradiol. **(A)** Macroscopic current recordings of TRPV1 channel from *X. laevis* membrane patches evoked by pulses of –100 to + 350 mV in 20 mV increments, with decreasing duration as potential increases, followed by a step at 190 mV to obtain the tail currents (*bottom*). 17-βE2: 17β-estradiol; 17-αE2: 17α-estradiol. **(B)** G/Gmax versus Voltage relationships generated from the tail currents and adjusted to a Boltzmann fit as follows: 1/(1 + exp(z(Vh-V)/RT)). 17β-estradiol produced a left shift of the G/V curve decreasing the V_0.5_ for activation from 131.6 ± 9.4 mV (*n* = 25, gray) to 46.2 ± 8.3 mV (*n* = 6, green). In contrast, 17α-estradiol produced a right shift of the G/V curve to 206.7 ± 18 mV (*n* = 7, blue). A bar plot showing V_0.5_ for each experimental condition is provided (*lower* panel). ***P < 0.0001 and **P < 0.001, non-parametric *t* test followed by Mann Whitney test. **(C)** Single channel recordings of TRPV1 exposed to different concentrations of 17β-E2. **(D)** Quantification of NPo for the experiment showed in **(C)**. NPo for control was 0.052 ± 0.009 (*n* = 3), 0.5 μM 17β-E2 increases NPo to 0.35 ± 0.05 (*n* = 3), NPo for 1 μM was 0.15 ± 0.018 (*n* = 2) and NPo for 5 μM was 0.21 ± 0.027 (*n* = 3). **(E)** Single channel recordings of TRPV1 exposed to 1 μM 17β-E2. **(F)** Quantification of NPo from experiment showed in **(E)**. Estimated NPo for control was 0.16 ± 0.023 (*n* = 5). When membrane patches were exposed to 1 μM 17α-E2 on NPo was 0.58 ± 0.017 (*n* = 4). Single channels were recorded at −100 mV.

In order to explore if 17β-estradiol can modulate TRPV1 activity, we measured TRPV1 currents in *Xenopus laevis* oocytes using the patch-clamp technique. The presence of 1 μM 17β-estradiol promoted a remarkable leftward shift in the conductance versus potential (G/V) relationships (Figure 2). The half voltage, V_0.5_, for activation shifted from 131.6 ± 9.4 mV to 46.2 ± 8.3 mV, revealing that TRPV1 can be directly activated by this steroid hormone. However, the stereoisomer 17α-estradiol did not increase TRPV1 activity. On the contrary, 1 μM 17α-estradiol rightward shifted the G/V curve to 206.7 ± 18 mV (Figures 2A,B). Moreover, an increase in TRPV1 activity induced by 17β-estradiol was also observed in single-channel recordings where the probability to find the channel open (*P*_*o*_) was significantly increased compared to controls when –100 mV was imposed to the patch membrane. 17β-estradiol NPo was: 0.052 ± 0.012 (*n* = 3; Control) and 0.355 ± 0.066 (0.5 μM 17β-estradiol; *n* = 3). This effect, however, was not reproduced by 17α-estradiol; 1 μM 17α-estradiol produced a NPo = 0.058 ± 0.017 (*n* = 5) a value similar to the control (Figures 2C–F).

### An Early Pulse of 17β-Estradiol Is Sufficient to Trigger TRPV1-Dependent Cell Protection

**FIGURE 3:**
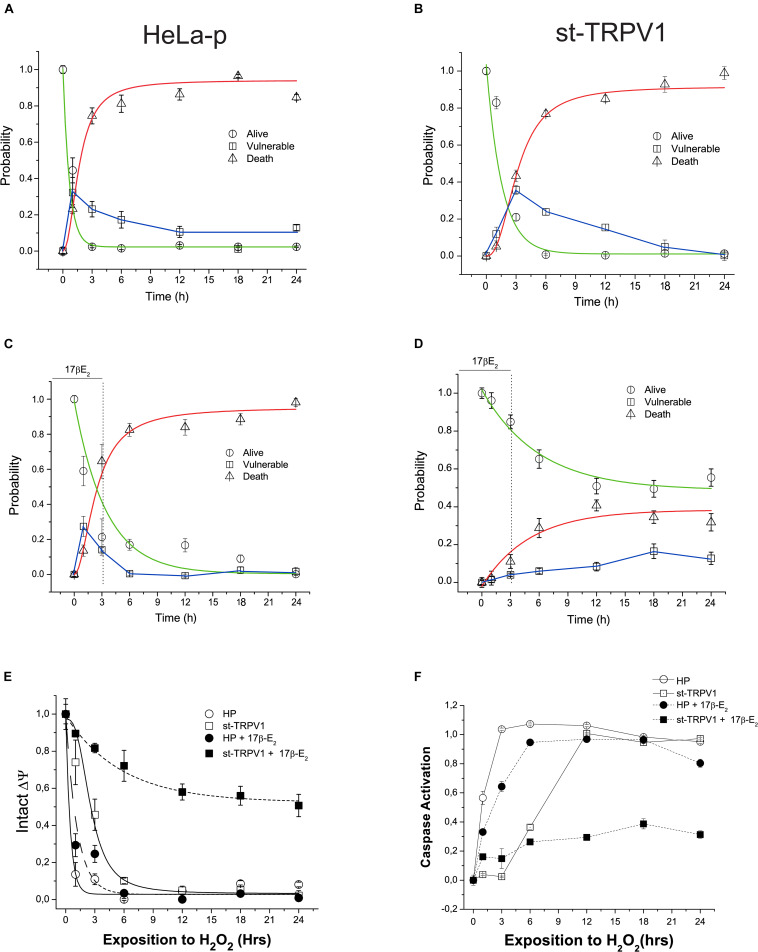
TRPV1 mediated 17β-estradiol improving of mitochondrial stability during oxidative stress. Time course of H_2_O_2_-induced cell death (1 mM) following a kinetic model of cell death in HeLa parental (HeLa-P) **(A)** and st-TRPV1 cells **(B)** (*N* = 9). **(C,D)** Effect of initial pulse of 17β-estradiol on time course of H_2_O_2_-induced cell death in HeLa Parental **(C)** and st-TRPV1 **(D)** cell line (*N* = 9). **(E)** Temporal course of ΔΨ in parental and st-TRPV1 cell lines measured by the rationometric probe JC-1. The data shows the first 3 h effect of 1 mM H_2_O_2_ and 17β-estradiol (*N* = 9). **(F)** Activation of caspase 3/7 by H_2_O_2_ in parental and st-TRPV1 cell lines in presence or absence of 10^–6^ M of 17β-estradiol at the first 3 h (*N* = 6).

We have studied the characteristics of the time-course of H_2_O_2_-induced cell death by performing a viability bioassay and kinetic recordings of the progression of the three-state cell model in parental and st-TRPV1 cells. Independently of TRPV1 expression, H_2_O_2_ exposure elicited almost complete cell death (Figures 3A,B). The analysis of vulnerable state indicated that H_2_O_2_ induced a vulnerable state via the loss of mitochondrial function in both cell lines, followed by plasma membrane disruption and cell death (Supplementary Figure S2). However, in st-TRPV1 we observed a delayed decrease in the alive state under H_2_O_2_ challenge compared to parental cells, and the peak of vulnerable state changed from 1 h for parental cells to 3 h for st-TRPV1 cells (Figures 3A,B), suggesting that TRPV1 expression is sufficient condition to improve cell viability under oxidative stress. Also, parental and st-TRPV1 cells were treated during the first 3 h of H_2_O_2_ challenge with a pulse of 1 μM 17β-estradiol. Parental cells showed similar results to those obtained without 17β-estradiol incubation (Figures 3A,C). Conversely, the exposure to 3 h 17β-estradiol was sufficient to induce cell protection against H_2_O_2_ cytotoxicity in TRPV1 expressing cell lines (Figures 3B,D). It is noteworthy that the preservation of the healthy condition was accompanied by the abolition of the vulnerable state peak. These data suggest that cells subjected to an initial pulse of 17β-estradiol became protected against oxidative cell death via a TRPV1 activated pathway.

### TRPV1 Mediated 17β-Estradiol-Improved Mitochondrial Function and Avoided Caspase Activation

**FIGURE 4:**
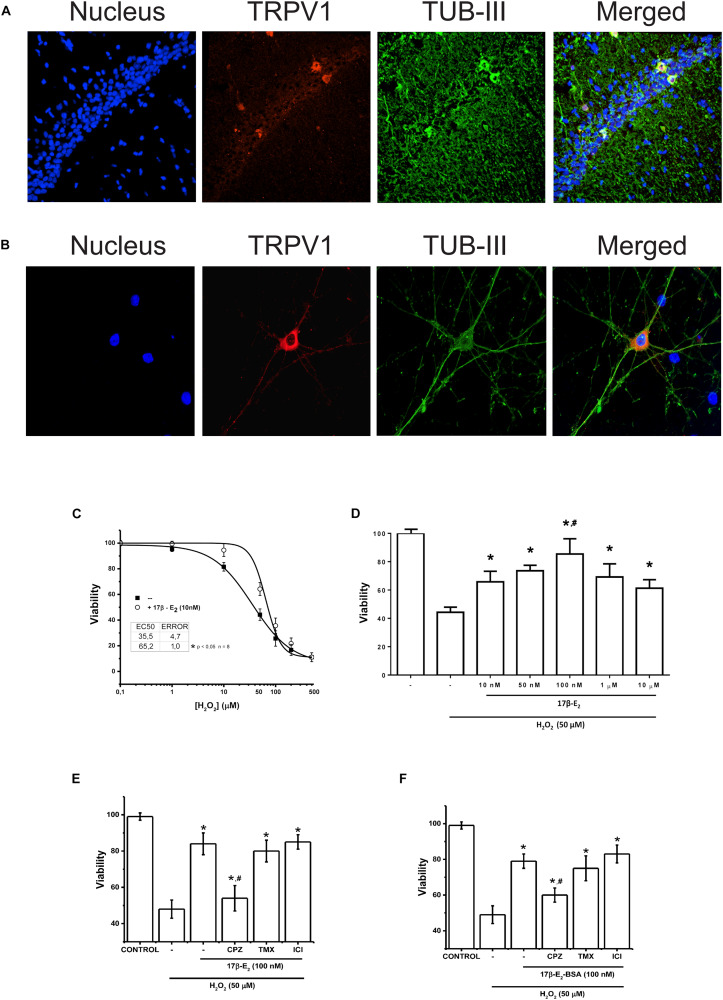
Membrane activity of TRPV1 is sufficient condition to run 17β-estradiol protection against oxidative stress. **(A)** Immunostaining for TRPV1 expression in rat hippocampus. The technique selectively detected the CA3 region of hippocampus. **(B)** TRPV1 detection in 7-day cultured hippocampal neurons. **(C)** Changes in cell viability after 24 h incubation with H_2_O_2_ at increasing concentrations in presence and absence of 17β-estradiol in primary culture of hippocampal neurons. **(D)** Bar graph summarizes the effect of increasing doses of 17β-estradiol over H_2_O_2_ 50 nM (*N* = 3) **(E)** Bar graph shows the effect of 10^–7^ M of 17β-estradiol on 5 × 10^–5^ M of H_2_O_2_ in hippocampus-derived neurons (*N* = 5). **(F)** The graph summarizes the effect of the impermeable adduct 17β-estradiol-BSA, 10^–7^ (17β-estradiol-BSA) on cell death induced by 5 × 10^–5^ M of H_2_O_2_ (*N* = 3). CPZ: capsazepine (10 μM); TMX, inhibitor of estrogen receptor α tamoxifen (10^–6^ M); ICI, inhibitor of estrogen receptor β ICI 182780 (10^–6^ M). Results are expressed as data normalized to untreated condition (UT) (without H_2_O_2_ or 17β-estradiol). Bars indicate means ± SD. Statistical differences correspond to one-way analysis of variance and Bonferroni’s *post hoc* test. ^#^*P* < 0.05 vs. 17β-estradiol CPZ; *P < 0.01 vs. 17β-estradiol H_2_O_2_.

To investigate the link between the TRPV1 activation by 17β-estradiol and mitochondrial function, we measured mitochondrial membrane potential (ΔΨ) using JC-1 probe. As shown in Figure 4E, exposure to 1 mM H_2_O_2_ reduced the mitochondrial membrane potential in both cell lines exhibiting the same delay in the decay as previously shown during the alive to vulnerable state transition (Figures 3A,B). However, a pulse of 1 μM 17β-estradiol preserved the mitochondrial function in st-TRPV1 parental cells (Figure 3E). The loss of ΔΨ could be related to the opening of MMP and the activation of caspases generally associated with apoptotic-like cell death by an intrinsic pathway. To determine the role of the activation of caspases in the H_2_O_2_ induced-cell death, we measured the time course of activation of total caspase (3–7) by flow cytometry in both cell lines compared with those obtained after 3 h exposure to 1 μM 17β-estradiol. We found that H_2_O_2_ induced the activation of caspases in both cell lines at different temporal windows. In parental cells, H_2_O_2_ exposure elicited the activation of caspase in the first hour (Figure 3F). However, at 3 h on 17β-estradiol we observed a reduction of caspase activity only in st-TRPV1. Overall, this evidence suggests that the activation of caspases induced by loss of mitochondrial membrane potential can be decreased by 17β-estradiol through an increase in TRPV1 activity. Altogether, our data suggest that TRPV1 is a membrane receptor of 17β-estradiol and that the protection against oxidative stress is related to the maintenance of mitochondrial function to preventing caspase activation.

### TRPV1 Mediated Membrane 17β-Estradiol-Induced Protection Against Neuronal Oxidative Stress

To corroborate our results, we tested the participation of TRPV1 in 17β-E_2_ cell protection in a primary cell culture derived from hippocampal neurons. Hippocampal neurons express functional TRPV1 and show intrinsic capacity to produce steroids and particularly estrogens (Mukai et al., 2006; Chávez et al., 2011). We confirmed the expression of TRPV1 in neurons only, both in hippocampus tissue and hippocampus-derived neurons by immunofluorescence, with highest expression in the cellular layer of CA1 region, mainly in cell bodies and dendrites (Figures 4A,B), confirming previous reports. Using cultured hippocampal neurons, we measured the effect of 17β-estradiol against oxidative stress produced by H_2_O_2_. We evaluated the cell viability of neuronal cells after 1 h of treatment with increasing concentrations of H_2_O_2_ in presence and absence of 17β-estradiol added 3 h before the oxidative insult (Figure 4C). Under all conditions, we observed a decrease in cell viability compared to controls and cell protection against H_2_O_2_ in those exposed to 17β-estradiol. Besides, the protective effect showed a biphasic component, with a peak of effect at 10^–7^ M and a decrease at micromolar concentration (Figure 4D). The half maximal effective concentration (EC_50_), was 52 ± 5 10^–6^ M. Using the EC_50_ for H_2_O_2_, we compared the effect of 17β-estradiol added 3 h before the oxidative insult. We found that the incubation of 10^–7^ M 17β-estradiol was sufficient to induce neuroprotection against H_2_O_2_ (Figure 4E). To evaluate the possible role of TRPV1 in cell protection, we blocked the channel activity with CPZ. We observed that, whereas CPZ significantly prevented the neuroprotective effect mediated by 17β-estradiol, tamoxifen and ICI 182780 (10^–6^ M) antagonists of estrogen receptors α and β, did not alter 17β-estradiol- induced neuroprotection (Figure 4E). Furthermore, 17β-estradiol-BSA produced the same protective effect as 17β-estradiol alone, suggesting that 17β-estradiol does not require diffusing through the membrane to exert its effect (Figure 4F). Altogether these data suggest that the expression of TRPV1 can mediate neuroprotection against oxidative stress, acting as a membrane receptor of the steroidal hormone.

## Discussion

This study demonstrates that 17β-estradiol is able to induce cell protection against oxidative stress through a mechanism dependent on TRPV1 activity. In addition, 17β-estradiol exposure prior to the oxidative injury only is sufficient to prevent H_2_O_2_-induced cell death. This level of control may be relevant in tissues that have the ability to aromatize androgens (Carreau et al., 2008; Zhang et al., 2014). For instance, mammalian glial cells do not produce 17β-estradiol under basal conditions. However, following brain injury and ischemia (Yague et al., 2008; Zhang et al., 2017) brain tissue expresses aromatase.

In general, estrogens are able to induce differential physiological effects through several mechanisms, some of them depend on the interaction with nuclear estrogen receptors whereas others may result from the activation of alternative estrogen-dependent routes with differential timelines. Here, TRPV1 expression was sufficient condition to confer 17β-estradiol-mediated protection against H_2_O_2_ challenge, independent of any intracellular classic receptor or plasma membrane diffusion. It has been reported that 17β-estradiol allows brain tissue protection, possibly by activating a voltage-dependent calcium channel (VDCC) (Wu et al., 2005; Feng et al., 2013). However, TRPV1-expressing HeLa cell lines exhibit 17β-estradiol-induced cell protection disregarding the expression of VDCC (Negulyaev et al., 1993).

In particular, estrogen can regulate TRPV1 activity and expression, playing a role in the sensitization of nociception (Cho and Chaban, 2012; Ho, 2013; Kumar et al., 2018). TRPV1 is differentially regulated by sexual steroids estrogen and testosterone, acting as positive and negative modulators, respectively (Chen et al., 2004). Our results show that 17β-estradiol-induced TRPV1 activity was not mimicked by its 17α-estradiol stereoisomer, suggesting that TRPV1 is able to discriminate between optical isomers with differential consequences. Moreover, the high lipophilicity of steroids raises the possibility that action mechanisms may occur through specific interaction with a protein inserted in the plasma membrane or by unspecific perturbation of lipid rafts surrounding the TRPV1 channel. 17β-estradiol may enhance TRPV1-mediated transient calcium influx in a stereospecific manner. In addition, the protection against oxidative stress obtained using 17β-estradiol-BSA, an impermeable probe which prevents plasma membrane diffusion, indicated that estrogen protection was initiated at the plasma membrane. This modulatory effect of 17β-estradiol has been previously reported with endogenous TRPV1 agonists in other tissues. For instance, 17β-estradiol enhances TRPV1-mediated vasorelaxation induced by CAP and anandamide (Ho, 2013). This role is particularly important considering that TRPV1 can integrate environmental physicochemical signals that are critical in controlling excitability and cell survival. Thus, integration of several signals may converge to improve cellular ability to deal with injury.

Similar to TRPV1, TRPM8 channel has been recently described as a testosterone receptor (Asuthkar et al., 2015). Both TRPV1 and TRPM8 are involved in pain nociception, inflammation and cell death, highlighting the therapeutic potential of determining the role of these channels in hormone related activities and cell stress injuries. Besides the wide distribution of TRPV1 channels, their ability to activate different response pathways according with the nature of the stimuli, their intensity or time pattern, support the idea that they are far more complex structures than sensory transmitters. We believe that TRPV1 has to be considered as a stress response protein. By integrating multiple signaling pathways, TRPV1 can modulate intracellular calcium levels to run the cellular response to stress and injury, a strategy that may underlie the implication of TRPV1 in glial, neuronal and cardiomyocyte death.

On the other hand, we propose that 17β-estradiol is also an inductor of stress response, because it is able to directly activate classical proteins involved in stress response such as heat shock protein (Stice et al., 2011) and also, because it exerts control of mitochondrial function by several means in oxidative environment. It is likely that both 17β-estradiol and TRPV1 are involved in oxidative stress survival response.

17β-estradiol may act as an acute or allosteric modulator of TRPV1 activity (Chen et al., 2004). It is well known that transient calcium influx is necessary to induce cell protection (Bickler and Fahlman, 2004; Feng et al., 2013). It is thus possible that TRPV1 mediates extra-nuclear action of steroids, similar to other TRP channels (Thiel et al., 2013; Wagner et al., 2008). This process must be rapidly activated in order to prevent calcium overload and loss of chemical potential energy (Feng et al., 2013). It can be regulated by transitory calcium signaling to establish direct or indirect coupling between the mitochondria and calcium waves (Malli et al., 2003; Zhao and Brinton, 2007). TRPV1 could act as a critical sensor that stimulates mitochondrial function in oxidative environment.

Actually, TRPV1 channels control the mitochondrial integrity through regulation of mitochondrial membrane depolarization in neurons (Medvedeva et al., 2008; Ramírez-Barrantes et al., 2018). For example, low doses of capsaicin in dorsal root ganglion trigger a transitory calcium signaling by TRPV1, which, in turn, activates calcium uptake by mitochondria and slow release (10–20 min later) to prolong glutamate release from these sensory neurons (Medvedeva et al., 2008). This particular ability to integrate environmental signals as steroid hormones and coupling with such vital organelles as mitochondria, could explain the opposite effects of TRPV1 described in the literature. We hypothesize that, at low concentrations, TRPV1 activators can induce beneficial effects on cell viability, i.e., the agonist CAP, preventing oxidative stress-induced cell death. High concentrations of activators or sustained activation of the channel might induce toxicity by deregulation of mitochondrial function. If TRPV1 is overactivated, a loss of transience of the signal is accompanied by dysfunction of mitochondria, calcium overload and cell death (Ramírez-Barrantes et al., 2018). It is likely that the physiological mechanism of action of TRPV1 (Pegorini et al., 2005; Huang et al., 2017) consists in, sequentially: activation, transient raise in intracellular calcium and consequent calcium-mediated desensitization. These regulated actions would allow preserving the integrity of mitochondrial function and cell viability. Estrogen, as suggested by the effect of a 3 h pulse, would produce a high-intensity initial signal activating TRPV1 with a large calcium influx, followed by an increase in mitochondrial calcium buffer capacity.

Further experiments are needed to clarify the specific steroid interaction with the TRPV1 channel and coupling of mitochondrial function. Considering that 17β-estradiol has been related to controlling the production of ATP, it is possible that cell protection is due to modulation of mitochondrial function. Estradiol can potentiate a cell protective pathway associated to functional coupling between TRPV1 activity and mitochondrial function in addition to other extra-nuclear estrogen actions described (Bao et al., 2011). The TRPV1 contribution to mitochondrial function needs to be further studied with focus on the coupling between TRPV1 activity and mitochondrial membrane potential.

Evidence that an initial pulse of 17β-estradiol is sufficient for cell protection is noteworthy. It is likely that H_2_O_2_ induces cell vulnerability through early mitochondrial failure, determining progression to cell death. Here, TRPV1 mediated 17β-estradiol ability to inhibit mitochondria depolarization thus preserving its function, diminishing the size of vulnerable population and decreasing cell death.

Furthermore, previous experiments of calcium imaging suggest that mitochondrial function is coupled to TRPV1-dependent intracellular calcium increase (Ramírez-Barrantes et al., 2018). We hypothesized that in the context of oxidative environment the possible mechanism of TRPV1-dependent protection could be mediated by a transient calcium increase leading to expand the mitochondrial calcium buffer capacity and cellular survival potential as has been previously suggested (Ge et al., 2016). Additionally, this transient calcium increase has been associated with a primary signal for expressing the anti-apoptotic protein Bcl-2 (Wu et al., 2005, 2011).

The role of mitochondria in cytosolic Ca^2+^ signaling has been related to calcium uptake and calcium buffering. Usually, when mitochondria are depolarized, the transient raise in cytosolic Ca^2+^ induced by different stimuli is larger (Vay et al., 2007) than in basal optimal mitochondrial conditions and inhibits the production of regenerative oscillations (Dedov et al., 2001; Vay et al., 2007; Medvedeva et al., 2008). This evidence indicates that mitochondria take up significant amounts of Ca^2+^ during cell stimulation and shows their vital role in regulating the calcium influx as a specific signal rather than a basic response to unspecific calcium overload. This function seems to be very important for many models of oxidative cell death, both mitochondria depolarization and calcium overload are present in cell death in glutamate cytotoxicity and ischemia reperfusion models (Nilsen and Diaz Brinton, 2003; Bickler and Fahlman, 2004; Wu et al., 2005). However, it has been reported that when calcium influx is triggered concomitantly with or just before the cytotoxic event, an increase in the mitochondrial calcium buffer capacity prevented cell death. This coupling activity between calcium influx and mitochondria is associated with a long-lasting conservation of the mitochondrial membrane potential due to the expression of anti-apoptotic protein Bcl-2 (Nilsen and Diaz Brinton, 2003). In fact, the calcium influx elicited by 17β-estradiol through VDCC, or even by ionomicin, is able to induce the active calcium uptake by mitochondria directly, but it relates also to the activation of MAPK pathway and AKT pathway to induce the expression of Bcl-2, inhibiting the mitochondria outer membrane permeabilization, and preventing the release of cytochrome c and the activation of caspases (Nilsen and Diaz Brinton, 2003; Bickler and Fahlman, 2004; Wu et al., 2005). In the same line, we suggest that the maintenance of mitochondrial function was able to diminish the activity of caspase enzymes necessary to induce apoptotic-like cell death via intrinsic pathways. It is possible that the membrane-associated action of 17β-estradiol through TRPV1 was able to induce cell protection not only by increasing the calcium buffering capacity but also by avoiding the activation of intrinsic apoptotic pathway. Nevertheless, the mechanisms of coupling between intracellular calcium influx, calcium buffer capacity and apoptosis are still to be clarified (Graphical Abstract).

We employed primary cultures of hippocampal neurons because they are responsive to estrogen and express TRPV1 endogenously (Chávez et al., 2011; Mukai et al., 2006). These neurons are very sensitive to oxidative environment. In these cells we demonstrated that 17β-estradiol and estradiol-BSA are capable of exerting protective effects, depending on a membrane receptor different from estrogen receptors α and β. In addition to classical TRPV1-expressing tissues, the channel appears at diverse locations in the context of oxidative injury. Our evidence that 17β-estradiol potentiates the activity of the channel by extra-nuclear mechanisms introduces important perspectives regarding the function of polymodal channels and of steroidal hormones (Ramírez-Barrantes et al., 2016). The study of the properties of TRPV1 may have several implications in cell physiology and therapeutic development. TRPV1 control of cellular response against oxidative environments to improve cell survival may lead to progress in stem cells graft, organ transplant, ischemia reperfusion disorders and neurodegenerative disease. TRPV1 have been used to control specific functions in neuronal and non-neuronal context, *in vitro* and in behaving animals (Huang et al., 2010; Stanley et al., 2012). In pancreas and neurons, the controlled activation of TRPV1 has been used to modulate responses as insulin secretion or producing fast activity onset. The interaction proposed in this paper points in the same line, being especially relevant in tissues that express TRPV1 and have the ability to produce relevant quantities of 17β-estradiol such as the gonads and brain. This could reveal new interesting routes for development of multidrug strategies on the basis of molecular discoveries conducted by clinical questions.

## Interest

The novel mechanism of 17β-estradiol may directly activate TRPV1-driven plasma membrane signaling coupled with mitochondrial function in a stereospecific manner, and it role to regulation of oxidative stress-induced cell death. This mechanism described in this study could generate molecular strategies for preventing oxidative stress-induced cell death, which occurs during neural-degeneration.

## Data Availability Statement

The datasets generated for this study are available on request to the corresponding author.

## Author Contributions

RR-B and PO conceived the experiments and drafted the work. RR-B, BR, CC, PD, KC, and KC-Z performed the experiments. RR-B, CC, CL, FS, PM, IM, KC, and PO analyzed the data. CL, KC, PO, and RL contributed reagents, materials, and analysis tools. RR-B, IM, PO, RL, and KC wrote the manuscript. All authors critically revised the manuscript for important intellectual content.

## Conflict of Interest

The authors declare that the research was conducted in the absence of any commercial or financial relationships that could be construed as a potential conflict of interest.

## Supplementary Material

FIGURE S1 TRPV1 overexpression in heterologous expression system. **(A)** Representative RT-PCR experiment showing TRPV1 mRNA expression (167 pb) for HeLa Parental (HeLa), HeLa st-TRPV1, Hippocampal tissue and transfection vector pCDNA3.1-TRPV1 plasmid. Housekeeping gene was s16 (102 pb). **(B)** Flow cytometry histogram showing TRPV1 protein expression by Immuno-FACS for HeLa Parental and HeLa st-TRPV1 and in presence of Anti-TRPV1. Negative control corresponds to the same technique in the absence of the primary antibody for TRPV1 (*n* = 4). **(C)** Immunofluorescence for TRPV1 by epifluorescence microscopy show distribution of TRPV1 protein in st-TRPV1 and parental HeLa cells.

FIGURE S2 Capsaicin and 17β-Estradiol protects against H_2_O_2_-induced cell death by preserving mitochondrial function in TRPV1 expressing cells. **(A)** Representative plots of the effect of CAP a TRPV1 agonist on H_2_O_2_-iduced cell death. **(B)** Bar graph summarizes the effect of CAP in cell death induced by H_2_O_2_. Bars show the mean ± SD (*N* = 9). **(C,D)** Representative dot-plot of temporal course of cell death from the kinetic model of cell death (Figure 3). **(C,D)** The data shows an initial phase of cell damage induced by H_2_O_2_ (1 mM) represented by the transition from alive (A) to vulnerable (V) state due to the collapse of mitochondrial function in both st-TRPV1 and HeLa-P, which eventually end in cell death for both cell lines. **(E,F)** However, after 3 h of 17β-Estradiol treatment only st-TRPV1 cells show a decrease in the number of vulnerable cells due to loss of mitochondrial function which in turns decrease the total number of dead cells (*N* = 9).

FIGURE S3 Effect on cell viability of pharmacological activators and inhibitors of estrogen receptor and TRPV1. Effect of 17β-estradiol E_2_, capzasepine (CPZ), tamoxifen (TMX), ICI-182 and hydrogen peroxide (H_2_O_2_) in st-TRPV1 cell viability measured by flow cytometry.

## Abbreviations

17α-E_2_/αE_2_: 17α-Estradiol
17β-E_2_/β E_2_: 17β-Estradiol
17β-E_2_-BSA/βE_2_-BSA: 17β-Estradiol conjugated with Bovine Serum Albumin
AVD: Alive-Vulnerable-Dead
CAP: Capsaicin
CPZ: Capsazepine
DMEM: Dulbecco modified Eagle medium
ERα: Estrogen receptor α
ERβ: Estrogen receptor β
FACS: Fluorescence activated cell sorting
FBS: Fetal bovine serum
H_2_O_2_: Hydrogen Peroxide
HP/HeLa-p: Parental HeLa cells
mRNA: Messenger RNA
NT: Non-treated
PI: Propidium Iodide
Q-PCR: Quantitative PCR
Rhod123: Rhodamine 123
ROS: Reactive oxygen species
RT-PCR: Reverse transcription PCR
shRNA: Short RNA hairpin
shRNA-SS: shRNA-Scrambled Sequence
st-TRPV1: Stable expression of TRV1
TRPV: Vanilloid transient receptor potential cannel
TMX: Tamoxifen
TUB-III: Beta Tubulin III
