# Supplementary figures and images for: TRPV1-Estradiol Stereospecific Relationship Underlies Cell Survival in Oxidative Cell Death
Source: Front Physiol. 2020 May 26;11:444. doi: 10.3389/fphys.2020.00444 (PMC7265966; doi:10.3389/fphys.2020.00444)

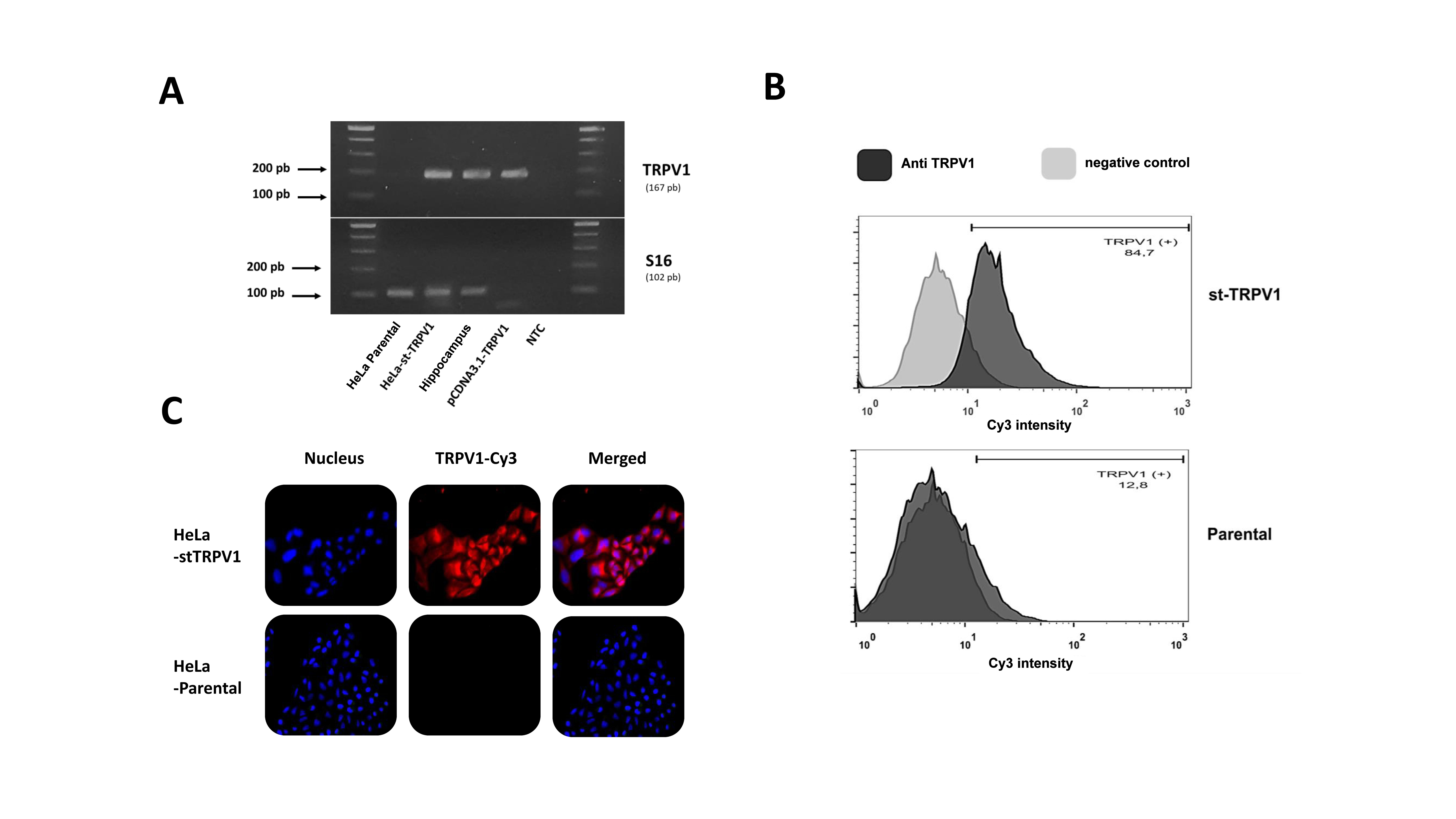

Supplement: FIGURE S1 — TRPV1 overexpression in heterologous expression system. (A) Representative RT-PCR experiment showing TRPV1 mRNA expression (167 pb) for HeLa Parental (HeLa), HeLa st-TRPV1, Hippocampal tissue and transfection vector pCDNA3.1-TRPV1 plasmid. Housekeeping gene was s16 (102 pb). (B) Flow cytometry histogram showing TRPV1 protein expression by Immuno-FACS for HeLa Parental and HeLa st-TRPV1 and in presence of Anti-TRPV1. Negative control corresponds to the same technique in the absence of the primary antibody for TRPV1 (n = 4). (C) Immunofluorescence for TRPV1 by epifluorescence microscopy show distribution of TRPV1 protein in st-TRPV1 and parental HeLa cells. [file Image_1.tif]

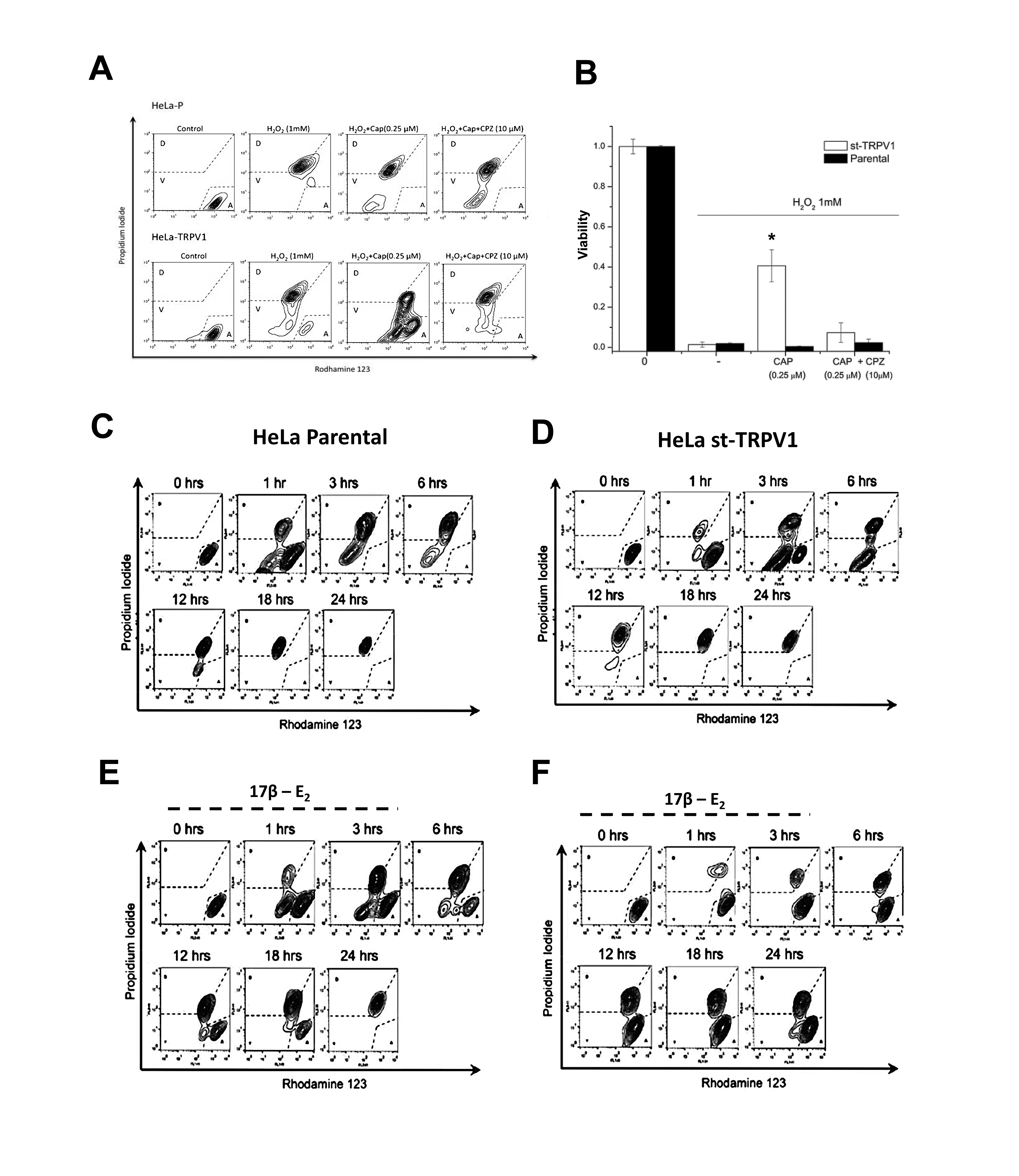

Supplement: FIGURE S2 — Capsaicin and 17β-Estradiol protects against H2O2-induced cell death by preserving mitochondrial function in TRPV1 expressing cells. (A) Representative plots of the effect of CAP a TRPV1 agonist on H2O2-iduced cell death. (B) Bar graph summarizes the effect of CAP in cell death induced by H2O2. Bars show the mean ± SD (N = 9). (C,D) Representative dot-plot of temporal course of cell death from the kinetic model of cell death (Figure 3). (C,D) The data shows an initial phase of cell damage induced by H2O2 (1 mM) represented by the transition from alive (A) to vulnerable (V) state due to the collapse of mitochondrial function in both st-TRPV1 and HeLa-P, which eventually end in cell death for both cell lines. (E,F) However, after 3 h of 17β-Estradiol treatment only st-TRPV1 cells show a decrease in the number of vulnerable cells due to loss of mitochondrial function which in turns decrease the total number of dead cells (N = 9). [file Image_2.tif]
